# Evidence use in E-cigarettes debates: scientific showdowns in a ‘wild west’ of research

**DOI:** 10.1186/s12889-021-10396-6

**Published:** 2021-02-16

**Authors:** Katherine E. Smith, Theresa Ikegwuonu, Heide Weishaar, Shona Hilton

**Affiliations:** 1 School of Social Work & Social Policy, University of Strathclyde, Lord Hope Building, 141 St James Road, Glasgow, G4 0LT UK; 2 MRC/CSO Social and Public Health Sciences Unit, University of Glasgow, Berkeley Square, 99 Berkeley Street, Glasgow, G3 7HR UK

**Keywords:** E-cigarettes, Vaping, Evidence, Policy, Scotland, Policy consultation, Interviews, Scientific controversy

## Abstract

**Background:**

Against a backdrop of declining tobacco use, e-cigarette markets are growing. The UK now has a higher percentage of e-cigarette users than any other European country. These developments have prompted fierce discussions in scientific, advocacy and policy communities about how best to respond. This article is one of the first to examine the role of evidence in these debates.

**Methods:**

We analysed 121 submissions to two Scottish policy consultations on e-cigarettes (in 2014 and 2015) and undertook interviews with 26 key informants in 2015–2016, following up with a sub-set in 2019–2020. All data were thematically coded, and our analysis was informed by insights from policy studies and the sociology of science.

**Results:**

First, we affirm previous research in suggesting that e-cigarettes appeared to have triggered a breakdown of old public health alliances. Second, we demonstrate that, amid concerns about research quality and quantity, actors are guided by normative outlooks (and/or economic interests) in their assessments of evidence. Third, we show that, despite describing e-cigarette debates as contentious and polarised, actors engaging in Scottish policy debates exhibit a spectrum of views, with most interviewees occupying an uncertain ‘middle ground’ that is responsive to new evidence. Fourth, we suggest that the perceived divisiveness of e-cigarette debates is attributed to recurrent media simplifications and tensions arising from the behaviours of some actors with settled positions working to promote particular policy responses (including by strategically enrolling supportive evidence). Fifth, we argue that the actions of these actors are potentially explained by the prospect that e-cigarettes could usher in a new tobacco ‘policy paradigm’. Finally, we show how scientific authority is employed as a tool within these debates.

**Conclusions:**

E-cigarette debates are likely to reconcile only if a clear majority of participants in the uncertain ‘middle ground’ settle on a more fixed position. Our results suggest that many participants in Scottish e-cigarette debates occupy this ‘middle ground’ and express concerns that can be empirically assessed, implying evidence has the potential to play a more important role in settling e-cigarette debates than previous research suggests.

**Supplementary Information:**

The online version contains supplementary material available at 10.1186/s12889-021-10396-6.

## Background

Electronic devices that mimic aspects of smoking (notably by delivering nicotine via inhalation) have been around since the late 1960s [1] but e-cigarettes have only achieved widespread use in the past decade [2, 3]. A 2017 survey found the UK had emerged as the European country with the greatest proportion (4.7%) of adults identifying as regular e-cigarette users [2]. The rapid growth of the e-cigarette market, combined with limited evidence of the consequences of their use, presents a range of challenges to public health policymakers and researchers [4]. Key questions include:

- Should e-cigarettes be banned (as consumer products that enable users to consume an addictive drug)?
- Alternatively, should e-cigarettes be promoted as a unique opportunity to support smokers who want to quit?
- If e-cigarettes are not banned, should they be regulated as tobacco products or medicines or something else and what does this mean, in practice?
- Will e-cigarettes contribute to a reduction in smoking?
- Will e-cigarettes sustain addictions and/or attract new (younger) consumers?
- As tobacco companies expand their market share of e-cigarettes, will they be able to regain policy credibility and influence or should Article 5.3 of the global Framework Convention on Tobacco Control apply in excluding them from e-cigarettes policy discussions?

In attempting to respond to these questions, public health researchers and policy actors are far from unified, with different policy bodies and states taking radically different approaches. For example, while Public Health England has promoted the relative safety of e-cigarettes (compared to conventional smoking), supporting calls for relaxed regulation (e.g. [5, 6]), the US has taken a more restrictive approach [7], banning most flavours from 1st February 2020 [8], while Australia and some US cities and states have opted to ban their sale entirely [9, 10].

It is not only policymakers who are divided: key charities involved in battling the tobacco epidemic have come down on different sides of e-cigarette regulatory debates [11]; media coverage suggests key actors promote opposing messages [12]; and public health advocates, once allies in the fight to reduce smoking, find themselves on opposing ‘sides’ [13]. Indeed, as e-cigarette use rises, the UK tobacco control movement appears to have transformed from a relatively unified advocacy-coalition, responsible for achieving major public health milestones [14], to a site of contestation and conflict [11, 13, 15]. Since a more divided tobacco control movement has long been an aim of transnational tobacco companies [16], it is crucial to understand how and why this fragmentation has occurred.

So far, the rapidly growing evidence-base does not appear to be resolving matters, at least in the UK. In Hawkins and Ettelt’s [13] assessment, two distinct ‘camps’ have emerged as evidence has entered the inevitably political space of policy discussions: a ‘harm reduction’ camp, which is broadly supportive of e-cigarettes and seeking relaxed regulation, and a ‘precautionary principle’ camp, which is concerned with the potentially negative consequences of e-cigarette growth, so pushing for more stringent regulation. Hawkins and Ettelt argue that, science and evidence are venerated as ‘a potential arbiter of policy disputes’ [13, p584], leading actors to claim that scientific consensus exists, despite the presence of alternative voices.

In analysing these debates, Hawkins and Ettelt [13] argue that Rein and Schön’s [17] concept of ‘policy frames’ helps explain how and why actors in both ‘camps’ are able to use evidence to support their positions; each set of actors frame the policy problem in contrasting ways and therefore ask different questions of the available evidence. Whilst one ‘camp’ focuses on the role e-cigarettes might play in reducing tobacco-related harms (seeking evidence regarding the relative harms of vaping compared to smoking), the other is concerned with the potential for e-cigarettes to act as a gateway into smoking for non-smokers, to re-normalise smoking-type behaviour, and to enable tobacco industry actors to re-enter research and policy debates. As a result, Hawkins and Ettelt [13] argue that evidence is unlikely to play a significant role in arbitrating e-cigarettes debates.

Accounts of the research and policy debates surrounding e-cigarettes in the UK [13, 15] contrast sharply with multiple earlier studies of the tobacco policy field, which frequently depict public health actors functioning as a relatively cohesive ‘advocacy coalition’ in the face of tobacco industry opposition [18–20]. Several of these older analyses used Sabatier and Jenkins-Smith’s [21] advocacy-coalition framework (ACF) to help explain their findings. The ACF provides a framework for understanding policy debates over time. It posits that networks of diverse actors (including policymakers, researchers, think tanks, journalists, interest groups and others) form opposing ‘advocacy coalitions’ that compete to influence ‘policy subsystems’. These ‘advocacy coalitions’ remain relatively stable over time because they form around ‘policy core’ beliefs which, in turn, reflect members’ ‘deep core’ beliefs (strongly held ontological and normative beliefs, such as a belief concerning the balance between individuals’ right to freedom versus social equality). From this perspective, while evidence may alter actors’ perceptions of the specific policy tools most likely to achieve their preferred outcomes, it is unlikely to alter a coalition’s views on what policy ought to be trying to achieve (i.e. ultimate policy goals) or broadly how this might be accomplished, since these beliefs are grounded in such deeply held values.

This paper draws on document and interview data to provide the first detailed empirical analysis of the role that evidence is playing in the contentious policy debates surrounding e-cigarettes in Scotland, a devolved UK nation with a particularly strong record on public health and tobacco control [22]. Theoretically, our analysis suggests that utility of the ACF in helping to explain tobacco control policy debates is diminishing in the context of e-cigarettes. Instead, we posit that combining Hall’s [23] concept of ‘policy paradigms’ and Gieryn’s [24] concept of scientific ‘boundary work’ together provide greater analytical insight, especially when it comes to understanding how scientific authority is employed within these debates. In the concluding discussion, we reflect on the implications of our findings for the likely future of e-cigarette debates in the UK.

## Methods

We conducted a thematic analysis of 121 publicly available documents and interviews with 26 key informants. The document analysis centred on written responses to the Scottish Government consultation on Electronic Cigarettes and Strengthening Tobacco Control in Scotland [25] and to questions on nicotine vapour products in the Scottish Parliament Health and Sports Committee Call for written views on the Health (Tobacco, Nicotine, etc. & Care) (Scotland) Bill [26]. These documents are publicly available (and we therefore identify the organisational authors where we cite these data).

**Fig. 1:**
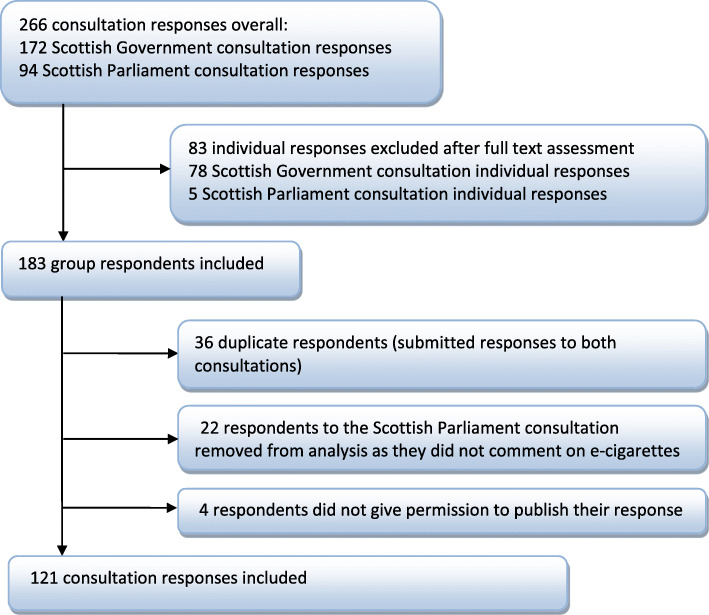
Flowchart of process for selecting documents to include in our analysis

Figure 1 provides a graphic depiction of the process of selecting 121 consultation responses for the documentary analysis. There were 266 consultation responses overall, but responses from individuals (largely vapers) were excluded in order to focus our analysis on the policy debates between organisational actors. In-depth thematic analysis of free text within the Scottish Government and Scottish Parliament consultation submissions was undertaken using NVivo11. Twenty consultation responses were initially coded independently by two members of the research team to ascertain policy preferences and references to (and claims about) evidence. This initial thematic coding was discussed by the full team to inform the final coding framework, which was then applied to the full document set by a single coder (the lead researcher: Theresa Ikegwuonu). The themes of most relevance to this paper were:

- Difficulties in researching e-cigarettes
- Research funding
- Sources of evidence
- Emerging evidence
- Quality of evidence
- Missing evidence
- Uncertainty of evidence
- Interpreting evidence
- Manipulating evidence
- Publishing
- Research communication or knowledge exchange work
- Discrediting or supressing discussion of evidence
- Same evidence used to support opposing views

To select potential interviewees from the consultation responses, we identified all organisations that had shown an interest in the process of negotiating and developing legislation to regulate e-cigarettes in Scotland. Interviewees representing these organisations were subsequently selected using purposive sampling to obtain a varied sample of stakeholders, representing a range of policy positions and organisation types. Potential interviewees were approached by email or telephone invitation (*n* = 47), via the project advisory board (*n* = 8) or via webchat and LinkedIn (*n* = 3). Up to three follow-up approaches were made. When participants agreed to take part, a suitable date and location for the interview was arranged. In total, we conducted 25 semi-structured interviews with 26 interviewees (one was a joint interview) between November 2016 and August 2017 (a generic version of the interview schedule is available as Additional File 1). Table 1 summarises interviewees, in terms of organisational sector and organisational positions on e-cigarette regulation (ascertained from consultation submissions).

**Table 1:** Overview of interviewees

Interview ID	Interviewee classification	Organisational position on key e-cigarette issues within consultation submission		
		Age of sale to under-18 s	E-cigarette advertising	Vaping in public places
1	Academic group	Restrict	Restrict	N/A^a^
2	Third sector/civil society organisation^c^	Restrict	Restrict	Do not restrict
3	Third sector/civil society organisation^c^	Restrict	Restrict	Do not restrict
4	Health service [NHS]	Restrict	Restrict	Restrict
5	Third sector/civil society organisation	Restrict	Restrict	Do not restrict
6	Government ^c^	N/A	N/A	N/A
7	Health service providers	Do not restrict	N/A	Restrict
8	E-cigarette industry^b d^	Restrict	Do not restrict	N/A
9	Academic group	N/A	Restrict	N/A
10	Health service providers	Restrict	N/A	Restrict
11	Academic groups ^c^	Restrict	N/A	N/A
12	Health service [NHS]	Restrict	Restrict	Restrict
13	Health service providers ^b^	Restrict	Restrict	Do not restrict
14	Health service providers ^b^	Restrict	Restrict	Restrict
15	E-cigarette industry^b^	Restrict	Do not restrict	Do not restrict
16	Retail	Restrict	Do not restrict	Do not restrict
17	Academic group	Restrict	N/A	N/A
18	Tobacco Industry	Restrict	Do not restrict	Do not restrict
19	Third sector/civil society organisation^c^	Restrict	Do not restrict	Do not restrict
20	Tobacco Industry ^b^	Restrict	Do not restrict	Do not restrict
21	Local authority with remit in health	Restrict	Restrict	Restrict
22	Third sector/civil society organisation	Restrict	Do not restrict	Do not restrict
23	Health service [NHS]	Restrict	Restrict	Restrict
24	Third sector/civil society organisation	Restrict	Restrict	N/A
25	Pharmaceutical manufacturer ^b^	N/A	N/A	N/A

^a^N/A indicates that no response was given in the consultation submission of the organisation represented by the interviewee. ^b^ Signals follow-up interview was conducted with the original interviewee from this organisation in 2019–2020. ^c^ Signals follow-up interview was conducted with a new interviewee from this organisation in 2019–2020 ^d^ Signals joint interview with two participants

All interviews were recorded and transcribed. We read and re-read the transcripts prior to the lead researcher (Theresa Ikegwuonu) developing a thematic coding frame in NVivo11, which was collectively discussed and agreed with the full team (in terms of analysing evidence use this framework largely mirrored the one used to code consultation submissions). We initially coded a sample of nine transcripts, all of which were cross-checked with a second team member, after which we discussed and refined the coding frame. The full set of transcripts were then systematically coded by TI using the revised coding frame, with all team members having access to the NVivo11 file. A second researcher cross-checked the coding on a sample of transcripts for consistency. Following data coding, the key themes of relevance to this paper were analysed by the lead author (who also read all transcripts in full, for context). The lead author then presented early analysis to the rest of the research team and to the project’s Advisory Board, developing this analysis further in response to feedback and discussion.

Conscious of the time that had passed since our data collection, and the speed with which e-cigarette products, markets and debates are evolving, in 2019, we approached all of our original interviewees with a request to undertake a follow-up interview. Between August 2019 and March 2020, we conducted follow-up interviews with all those who were available (see Table 1), using a follow-up interview schedule (Additional File 2). This included representatives from eleven of the original organisations in our interview sample (seven were individuals from our original set of 26 participants, two of whom gave a joint interview at both stages; five were new interviewees, based at an organisation in our original sample). This paper primarily focuses on our original data set but the final section of our results briefly summarises how follow-up interviewees suggested debates had evolved since the original data collection.

This study was reviewed by and obtained ethical approval from the University of Glasgow’s Social Sciences Research Ethics Committee.

Finally, given the contested nature of e-cigarettes debates, we feel it is important to acknowledge our own positionality, before presenting our analysis. As researchers, we are all committed to improving public health and reducing health inequalities but none of us has yet reached a clear or fixed position regarding the potential impact of, or optimal regulatory approach to, e-cigarettes. Several of us have previously published research that critically examines tobacco industry influence on public policies and this has left us cautious about the potential for transnational tobacco companies to regain public and policy influence via e-cigarette investments.

## Results

The results are organised into six sub-sections, ordered to help convey the complex and nuanced narrative that emerged from our analysis. We use the term ‘actors’ to refer collectively to our interviewees and the authors of the policy submissions we analysed.

### Public health fighting amongst itself: the emergence of e-cigarettes ruptures an old alliance

In line with the existing literature, both the interviews and consultation submissions provide copious evidence of the contested nature of e-cigarettes in Scotland, with interviewees describing debates as ‘nasty’, ‘unpleasant’ and ‘horrible’ (see also [15]), and one interviewee claiming that some participants ‘almost feel bullied into silence’. In short, as the following three extracts attest, our data suggest that the growth of e-cigarettes in the UK (which has introduced a range of new actors) has caused a long-standing tobacco control advocacy-coalition to rupture, with the consequence that previously positive, collaborative relationships have broken down:

Interviewee 11 (academic researcher): *“The […] criticism that we get nowadays is actually from our colleagues, it’s from public health, and there are particular individuals, [gives named examples], who have been incredibly critical of the position that [we and others] have taken. They’ve been vicious. They’ve been insulting. […] So, it’s hugely divisive and I would say that relationships that used to be very positive – so I used to have a very positive relationship with [named example] and that’s not the case anymore – […] now it’s public health fighting amongst itself instead of what we used to do, which is we knew who the enemy was, the enemy was the tobacco industry and that has… the debate has shifted.”*

Interviewee 12 (NHS – National Health Service): *“It’s been a terrible shame actually because having worked in tobacco control for the twenty years […], I have never known an issue to cause the divisiveness that this has caused. […] I mean, I got some feedback on something which was in an extremely unprofessional manner actually. I was absolutely horrified. And academics who I have worked with very closely and very well with, in the past.”*

Interviewee 14 (pharmacy): *“We tried to do a joint statement with [a public health organisation] in the beginning but then we realised that, actually, we’re coming from two different places. […] Before e-cigarettes, we would’ve done a joint statement with [that organisation] very easily because we’re very much on the same page. We suddenly realised we’re actually on quite different pages on this, so we haven’t done anything.”*

In Sabatier and Jenkins-Smith’s [21] accounts of the ACF, the values around which coalitions form are depicted as so deeply embedded that they are hard to shift. Yet, our data suggest that the public health coalition broke down relatively easily in the context of e-cigarettes. This suggests that analyses of the tobacco policy subsystem prior to the growth of e-cigarettes may have implied a greater degree of normative coherency than was necessarily the case [27].

### Negotiating the ‘wild west’ of evidence with values, beliefs and interests

In explaining how they were negotiating the new tobacco policy landscape, almost all our interviewees suggested that evidence had an important role to play. However, there was widespread agreement that available evidence was limited and not always of high quality:

Interviewee 19 (pro-e-cigarette campaign group): *“The standard of some of the evidence, and to be fair on both sides, is not great quality...”*

Interviewee 4 (NHS): *“The evidence is absolutely rubbish. There’s stuff published which is terrible, awful studies. People over-egging stuff. People, you know - it is a kind of Wild West with e-cigarettes, with people making all sorts of claims about it.”*

Interviewees generally attributed the limited quality of available evidence to the novelty of e-cigarettes, the diversity of products captured by the ‘e-cigarettes’ label and the dynamism of both the market and products, with several interviewees highlighting the inevitable absence of long-term evidence. For example:

Interviewee 19 (pro e-cigarette campaign group): *“The evidence that’s missing is about long-term use, and the consequence and risks associated with that. And the reality is you can’t have long-term evidence until the product’s been around for a long time.”*

Interviewee 2 (Third sector health advocacy group): *“You just can’t generalise about this huge range of devices and then huge range of fluids as well. It’s not a thing, and that’s what I’m always trying to remind enquirers, it’s just, it’s not one thing (sigh). […] I can’t think of any product that’s quite as diverse.”*

Despite this consensus, most actors appeared to have preferred policy positions on key regulatory issues and most employed evidence to support these positions (this was true of both interviewees and the organisational consultation responses). In the following extracts, we see both interviewees attributing contrasting positions on e-cigarettes to pre-existing views and values:

Interviewee 5 (Third sector/health charity): *“I think some academics have been so keen to actually gather evidence which supports their view, rather than gathering the evidence and letting that form their view, and I have to confess, I’ve been a bit shocked at the extent to which I think I’ve seen that take place.”*

Interviewee 1 (academic researcher): *“[I]f your basic premise is inconsistent with use of electronic cigarettes… because you believe that nicotine addiction is wrong or because you believe that they are a force of evil… driven by the tobacco industry, then you are likely to take an extreme view in interpreting evidence that supports those positions.”*

Indeed, some of our interviewees openly reflected that, having settled on a particular policy position, they had then sought evidence to support it. For example:

Interviewee 1 (academic researcher): *“We’re all guilty of it to some degree, of using evidence to support an argument rather than basing arguments on evidence.”*

Interviewee 14 (pharmacy sector): *“I was trying to find out more on that side of it, hoping for a few golden nuggets […] which would support…what we were trying to say.”*

In sum, our data make clear how, in the face of uncertain evidence, actors draw on their beliefs and normative values to help them make sense of the evidence that is available. This finding is in line with Sabatier and Jenkins-Smith’s [21] claim that ‘values’ and ‘beliefs’ shape policy debates and inform emerging coalitions. Up to this point, our findings appear to reinforce Hawkins and Ettelt’s [13] claim that evidence is unlikely to be an effective arbitrator in the debates surrounding e-cigarettes.

### The plot thickens as a hidden ‘middle ground’ emerges

So far, our analysis tells a very similar story to that of Hawkins and Ettelt [13]: the emergence of e-cigarettes disrupted old alliances and new divisions emerged as actors approached the issue (and the evidence) with contrasting normative outlooks (or ‘frames’). Yet, in our data, this narrative only seems to capture the actors who had settled on clear policy positions about e-cigarettes. We were surprised (given the clear sense of divisiveness that interviewees recounted) to find that our analysis suggested many participants had not settled in one or the other of these ‘camps’ and instead self-identified as occupying a ‘middle ground’. Reflecting this, the following interviewee said they felt this was where the majority of participants in e-cigarette debates (within Scotland) were positioned:

Interviewee 1 (academic researcher): *“There are some who want very high levels of regulation and some who feel that we shouldn’t have any regulation at all […] But […] those are extremes on a spectrum, and I think most people want proportionate regulation that protects consumers but makes sure the products stay available.”*

Although this claim may, as another interviewee suggested, be partially explained by the strategic value of self-positioning away from the extremes of a debate, it nonetheless speaks to the fact that many of our interviewees were resistant to being positioned in either of the two, opposing ‘camps’ that Hawkins and Ettelt [13] identified. Moreover, both our documentary and interview data make clear that there were areas of consensus regarding the regulation of e-cigarettes in Scotland (e.g. actors generally agreed on the need for age-of-sale restrictions and, at least partially, on the need to regulate advertising while only a much smaller number of participants supported restrictions/bans on vaping in public places [11]).

**Fig. 2:**
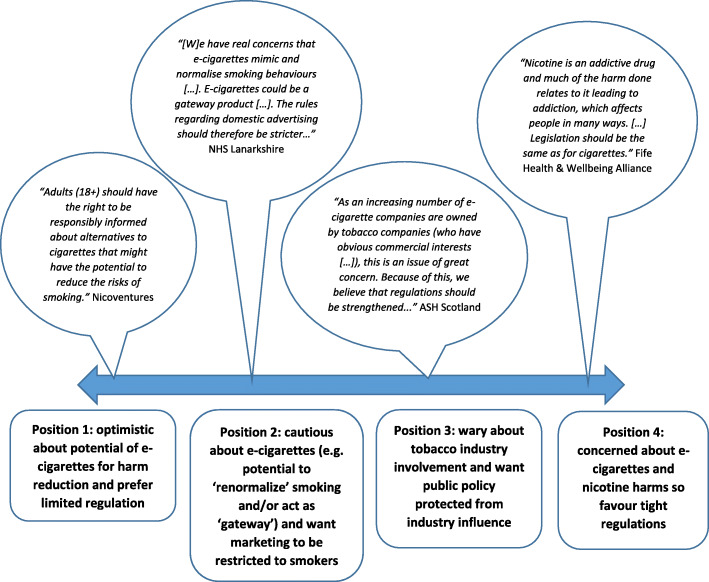
The spectrum of actors’ positions on e-cigarettes

Overall, our analysis identified a range of views about e-cigarettes within our data, each of which was associated with distinct policy positions. As Fig. 2 depicts, these views fall along a spectrum. At one end, there are those whose beliefs and values (and, in some cases, economic interests) meant they expressed optimism about the impacts of e-cigarettes and favoured limited regulation (Position 1 in Fig. 2). At the other end, there were those who viewed e-cigarettes as a mechanism to maintain nicotine addictions and renormalise smoking, who wanted e-cigarettes to be regulated similarly to traditional tobacco products (Position 4 in Fig. 2). Unsurprisingly, those with an economic interest in e-cigarettes generally expressed views that place them clearly in Position 1 (Fig. 2).

Beyond this, however, our data suggest few actors could be clearly positioned at either end of the spectrum. Rather, most fell somewhere in the middle, favouring some degree of regulation but varying in their perspective on specific regulatory proposals. For these interviewees, the position their organisation adopted on specific regulatory proposals in the consultation was often presented as evidence-informed and open to change.

Within this ‘middle ground’, we identified two dominant positions. The first (Position 2) involved caution about e-cigarettes on the basis of the *potential harm of the products*, mainly in terms of the potential for e-cigarettes to act as a ‘gateway’ into smoking for non-smokers and youth but also in terms of renormalising smoking. The difference between Position 2 and Position 4 is that Position 2 was not informed by a belief that nicotine addiction is, in principle, undesirable but about concerns with e-cigarette products that could more easily be empirically assessed. In contrast, Position 4’s concern with nicotine addiction effectively ruled e-cigarettes as undesirable by virtue of the fact they are largely designed to deliver nicotine to users. Position 3 is placed closer to the right-hand side than Position 2 as it is underpinned by a principle of tobacco industry exclusion that suggests less openness to limited regulation than Position 2.

Importantly, our data suggest that it was not always possible to place actors at specific points along the spectrum (Fig. 2). Rather, some consultation responses and interview transcripts cut across Positions 2 and 3, which makes sense when we consider the underlying normative outlook associated with the different positions. While Positions 2 and 3 are distinct from one another (and not interdependent), they are also compatible. In contrast, the normative outlooks underpinning Position 1 (harm reduction should be prioritised) and Position 4 (addictive consumption products should be tightly regulated) make these positions incompatible, regardless of any insights provided by evidence. Only a small number of actors occupied Position 4, while many of the actors whose views placed them clearly at Position 1 (Fig. 2) had material economic interests in the e-cigarette market. Actors occupying Positions 2–4 all supported greater regulation of e-cigarettes than actors at Position 1, with the strength and extent of desired regulations increasing with position number (i.e. those favouring the strictest regulation occupied Position 4).

All this suggests that, while positions within e-cigarette debates are shaped by normative outlooks and material interests, many actors’ positions are more fluid and intersecting than Hawkins and Ettelt [13] suggest, making the potential for alliances more complex. Crucially, while Hawkins and Ettelt [13] were pessimistic about the potential for evidence to play a role in arbitrating e-cigarette debates, our data suggest that research can inform (and, indeed, has informed) actors’ policy positions, especially for actors occupying the uncertain ‘middle ground’ (Positions 2 and 3 in Fig. 2). In the following extract, for example, an interviewee reflects that their organisation has shifted from Position 2 towards Position 1, in light of emerging evidence:

Interviewee 10 (health service provider): *“Going back to where we started to look at this, one of the biggest concerns seemed to be about children and young people using these products as a gateway. And we’ve monitored the data very closely and talked to other groups […] and it’s very obvious that the data in the UK doesn’t suggest that’s a problem, and consistently so. […] So where I think in the past we were certainly talking about regulating [e-cigarettes] very strongly to ensure the gateway effect didn’t happen, we’re very clear now that the evidence and data just has… there’s probably enough years of it and it hasn’t shown that to be a big issue.”*

Actors whose economic interests were intertwined with the success of e-cigarettes and those whose normative outlooks placed them firmly at either end of the spectrum in Fig. 2 seem unlikely to adapt their position in response to evidence. However, given most of the actors in our sample were positioned in the more uncertain ‘middle ground’, and most referred to research as meaningful, our data suggest that evidence could play an important role in the evolution of e-cigarettes policy debates.

Theoretically, our analysis in this section suggests that the e-cigarettes market has introduced complex dynamics to the tobacco policy and advocacy field. As a consequence, Sabatier and Jenkins-Smith’s [21] widely employed ACF no longer seems like a sufficient theoretical tool for understanding tobacco policy debates since, rather than two clear ‘advocacy coalitions’ with distinct values, our analysis suggests there are at least four distinct positions, informed by a complex interaction of values, interests and uncertainties. We return to and develop this analysis in our concluding discussion.

### Caught in the crossfire and forced to choose sides

Since our analysis suggests that many of the actors engaging in Scottish policy debates about e-cigarettes occupy a fluid and uncertain ‘middle ground’ (Fig. 2), this raises questions as to why our interviewees ([and others: [15]) describe e-cigarette debates in the UK as so divisive and antagonistic. In our data, interviewees suggested not that most actors had chosen ‘sides’ but rather that it was difficult to avoid being positioned by others as being on one or the other ‘side’ of e-cigarette debates. In the following extract, for example, an interviewee reflects that they felt even the act of seeking academic advice could lead others to (mis)position the advice-seeker:

Interviewee 12 (NHS): *“I just do not want involvement with being perceived to be on one side or the other […] which makes it slightly difficult because where I would probably naturally want to involve academics in certain issues [… but] if you get involved with one academic, then the perception is, that’s your approach. When actually, it’s not.”*

Another interviewee – an academic who said they tried to speak to ‘both sides’ - was concerned about how they might be represented in our research as a consequence of this.

Interviewees attributed the difficulty in avoiding being positioned on one or the other ‘side’ to two sources: (i) mass media coverage of e-cigarette debates; and (ii) the behaviour of other actors. On the first of these, multiple interviewees noted the large appetite for stories about e-cigarettes in the UK media and said they felt stories (and sometimes university or journal press releases) tried to position everyone as being in favour or against e-cigarettes (a perception that analysis of UK newspapers supports –[7]). For example:

Interviewee 10 (Health Professional Body): *“It’s at times been particularly divisive and not always a hundred percent clear why, and […] actually, sometimes the positions of organisations aren’t as far apart, when you look at the detail, as they might seem. When you have people looking for a high level kind of position or you get, again, media reports of your position, they tend to focus on the extreme aspects or the kind of overarching headlines, rather than the detail, and I understand that, but I certainly think it’s been a real issue on this area.”*

Interviewee 1 (academic researcher): *“[I]n studies of children’s use and gateway progression […] the evidence published to date doesn’t support that that is happening to any significant degree. But that’s not what you would think, […] reading the press releases that go with papers.”*

The second explanation interviewees gave for the difficulty they faced in avoiding being positioned at either end of the spectrum related to behaviours and actions of some of the other actors (including academics) engaging in e-cigarettes debates. As the opening section of our results make clear, some interviewees described other actors in ways that suggested they were working to attract support for their preferred position, while undermining opposing positions. Here, the interview data were at their most critical. While one interviewee described the situation as ‘healthy debate’, several others expressed frustration, surprise, exasperation and annoyance at the behaviours they had witnessed and heard about (see also the extracts in the opening section of the results):

Interviewee 12 (NHS): *“[T]he examples of what I have seen […] and what I’ve heard in terms of reports of events and the way people have been treated if they don’t subscribe to a particular line. I’ve also had a look at, well I did and I haven’t gone back to look at it again, because I was so horrified at what I saw, but just some Twitter accounts of the latest research that has come out about things and […] just comments around those issues and, yeah, it’s shocked me.”*

The claim that media interest in e-cigarette debates served to unhelpfully simplify debates by positioning actors as ‘for’ or ‘against’ e-cigarettes is unsurprising since it is in line with existing analyses of mass media coverage of other scientific debates (e.g. [28]). However, the claim that key actors, including academics, were working hard to attract others to one side or the other requires further explanation.

### High stakes as a ‘policy paradigm’ shift looms

Our interviewees did not generally proffer their own explanations as to why they felt some actors were working to attract others to particular ends of the spectrum (Fig. 2) or why, as described at the start of the results and elsewhere [15] e-cigarettes debates seemed to be associated with higher than usual instances of emotive, sometimes unpleasant, interactions. Our analysis here is therefore a little more speculative but the data do suggest that at least some interviewees believed there was a great deal at stake, variously describing the need to get the regulatory approach ‘right’ as ‘crucial’ and ‘vitally important’. We can also see examples of interviewees reflecting on the possibility that previously accepted tenets of tobacco control policy may shift in the context of e-cigarettes. For example, in the following extract, an interviewee muses whether the emergence of e-cigarettes may require a change to the public health consensus around excluding tobacco industry interests from research and policy discussions:

Interviewee 1 (academic researcher): *“It becomes difficult. So suppose… British American Tobacco were to buy Johnson & Johnson nicotine replacement therapy range… and became one of the biggest suppliers of medicinal nicotine in the UK, how would we react then? And that, so there would need to be some sort of change in the way that we dealt with tobacco industry in those circumstances. At the moment, the tobacco industry, or until now, has made it very easy for us to portray them as evil and […] organisations that we shouldn’t have anything to do with because they haven’t produced any products that we actually want. But whether they’re in that situation now is far from clear...”*

Several interviewees, including the one quoted above, questioned whether tobacco control may need to alter its approach to excluding commercial interests, given the tobacco industry’s new role in e-cigarettes. In contrast, others emphasised some of the successes of tobacco control to date, noted previous efforts by tobacco industry actors to prevent and undermine tobacco control efforts, and stated they felt there remains a clear need to protect public health from tobacco industry influence (see Position 3 in Fig. 2). This division speaks to Studlar and Cairney’s [29] analysis of tobacco policy development, in which they propose that the current ‘policy paradigm’, which views tobacco as a ‘social and global menace’, may soon evolve. While one possibility would be for the current paradigm to transition into a ‘neo-prohibitionist’ paradigm, in which further and stronger tobacco control policies are pursued, they also identify a possibility of a more ‘radical’ shift to a ‘harm reduction’ policy paradigm.

The concept of a ‘policy paradigm’ comes from Hall [23] who, employing economic policy as a case study, showed that policymakers tend to work within particular interpretative frameworks of ideas that he called ‘policy paradigms’:

‘Like a *Gestalt*, this framework is embedded in the very terminology through which policymakers communicate their work, and it is influential precisely because so much of it is taken for granted and unamenable to scrutiny...’ (Hall, 1993, p279).

Viewed from this perspective, policy responses to the growth of e-cigarettes in the UK could involve one of three options: (i) the current policy paradigm continues and e-cigarettes become part of the existing ‘smoking cessation toolbox’ (interviewee 14 – pharmacy); (ii) e-cigarettes are tightly regulated, possibly banned, as policy shifts to a ‘neo-prohibitionist’ paradigm; or (iii) e-cigarette growth continues and informs a shift to a new ‘harm reduction’ policy paradigm (Studlar and Cairney, 2014). In our data, there was very little evidence of actors working to actively promote a shift towards a ‘neo-prohibitionist’ policy paradigm (although some suggestions for a tobacco control 'end game' do posit this kind of shift, e.g. [30]). However, it was clear that some interviewees were openly identifying perceived failures of existing policy approaches; a development that Hall (1990) argues forms an important component of work to establish a new policy paradigm:

Interviewee 25 (industry manufacturer association): *“If you look at the smoking rates… they are going down which is good, but it’s a really big health inequalities issue, and the people in the kind of poorer, more deprived areas have much higher smoking rates than the people in the more affluent areas. So whilst the overall trend is down, I think that there’s a lot still that needs to be done to support those people that are still smoking to become free of that addiction.”*

Some interviewees, such as the following, explicitly used claims about the failure of current approaches as a rationale for considering new approaches:

Interviewee 1 (academic researcher): *“I’ve been promoting harm reduction as an alternative strand or a complementary strand to tobacco control ‘cause we aren’t doing enough for people who are addicted to tobacco. We’re doing quite well at preventing smoking uptake but we’re not doing very well at helping established smokers to quit. And harm reduction is an opportunity to do that.”*

**Fig. 3:**
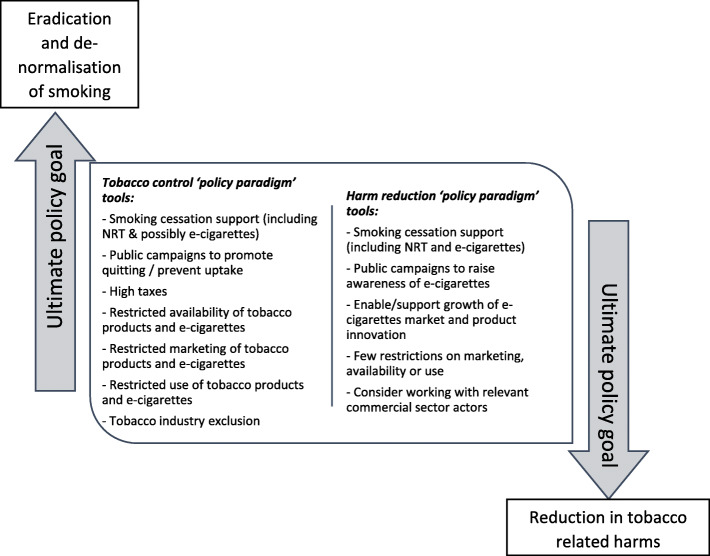
Two competing ‘policy paradigms’ in the tobacco field

To explore this further, Fig. 3 summarises the two ‘policy paradigms’ that were most evident in our data: the existing ‘tobacco control’ paradigm and a potential ‘harm reduction’ alternative, which was actively being promoted by several interviewees and consultation respondents. At this stage, it is far from clear that a new ‘harm reduction’ policy paradigm will emerge. However, the extracts in this section are illustrative of the fact that some of our interviewees suggested that we *may* be on the cusp of significant changes in the way we think about tobacco policy.

From the perspective of the ‘policy paradigms’ literature it is unsurprising that e-cigarettes debates feel divisive since, at least for those clearly favouring one ‘paradigm’ over the other, the stakes are very high. Where potential policy paradigm shifts emerge, Hall [23] suggests that advocates of competing policy paradigms are unlikely to agree on a common body of evidence (or even data) to adjudicate, making sociological and political dimensions crucial to understanding how debates evolve. Within this, Hall argues that ‘issues of authority will be central’, meaning the ‘policy community will engage in a contest for authority over the issues at hand’ [23]. Unsurprisingly, for an issue so core to public health, our data suggest contests for authority in e-cigarette debates are centred on contrasting claims to *scientific* authority, even though these debates are often entangled with more normative divisions (notably around the appropriate role of tobacco industry actors in policy discussions).

### A scientific showdown ensues

Reflecting the importance of scientific authority for public health, we found scientific evidence regularly being enlisted by actors to support their positions. Indeed, for each e-cigarette regulatory issue, we can find submissions expressing directly contrasting preferences, each citing supporting evidence. The following two extracts - responses to consultation questions about restricting vaping in enclosed public places - are illustrative:

“Recent research suggests that the second-hand smoke from some e-cigarettes may contain higher levels of certain harmful metals than in traditional tobacco cigarettes […]. Coupled with this, the World Health Organisation has already proposed banning the use of e-cigarettes indoors as they increase the background level of toxicants and nicotine. Children in Scotland believes that in light of this evidence that there should be further regulation of e-cigarettes.” (Children in Scotland).

“E-cigarette vapour does contain toxicants however this is usually at levels which are far lower than those found in tobacco cigarettes. […] The consensus is that any harm from e-cigarettes is likely to be far less than that from tobacco. Therefore, we believe that there is currently insufficient evidence to justify a ban on the use of e-cigarettes indoors and in enclosed public spaces on the basis of harm from second-hand vapour.” (Cancer Research UK).

For the most part, where this occurred, participants either appeared to be drawing on different sources of evidence or framing the implications of the same evidence in contrasting ways due to the normative lens through which they were interpreting it. However, we also found occasional examples of evidence being actively misrepresented. For example, a consultation submission from Totally Wicked Ltd. claimed that research undertaken by John Moores University demonstrated that there is no correlation between electronic cigarette advertising and use amongst children when, on checking the original qualitative research [31], we found that it did not assess correlation between exposure to advertising and use. Decisions to misrepresent research as supporting a particular position speaks to the importance that those involved in e-cigarettes policy debates attach to being able to claim positions are evidence-based.

**Fig. 4:**
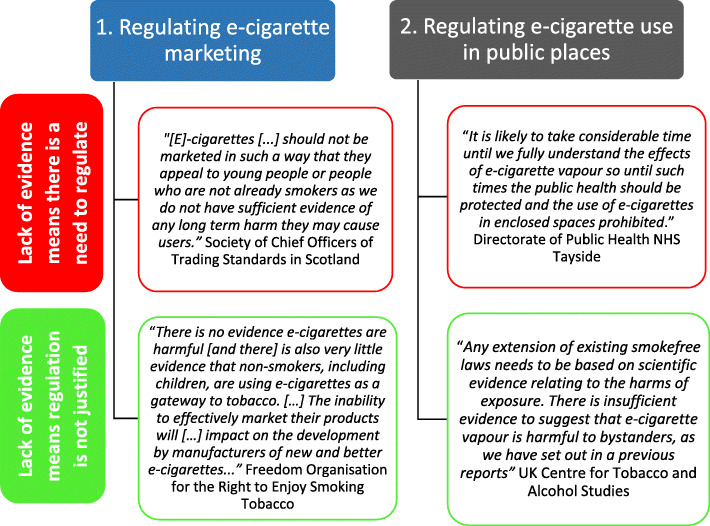
Illustrative examples of the way in which some actors used a lack of evidence to support opposing regulatory positions

Interestingly, where actors acknowledged that evidence was lacking, our data provide examples of this absence being employed to reach directly opposing conclusions, as Fig. 4 illustrates. Although public health is, as a discipline and area of practice, strongly committed to the idea of evidence-informed decision-making, Fig. 4 highlights that early evidence in contentious policy areas (or the associated lack of evidence) is not necessarily a tool that can easily settle policy controversies. Indeed, as Table 2 illustrates, the data are replete with examples of participants affirming the scientific credibility of their preferred position and, in some cases, discrediting the scientific credentials of those with alternative positions.

**Table 2:**
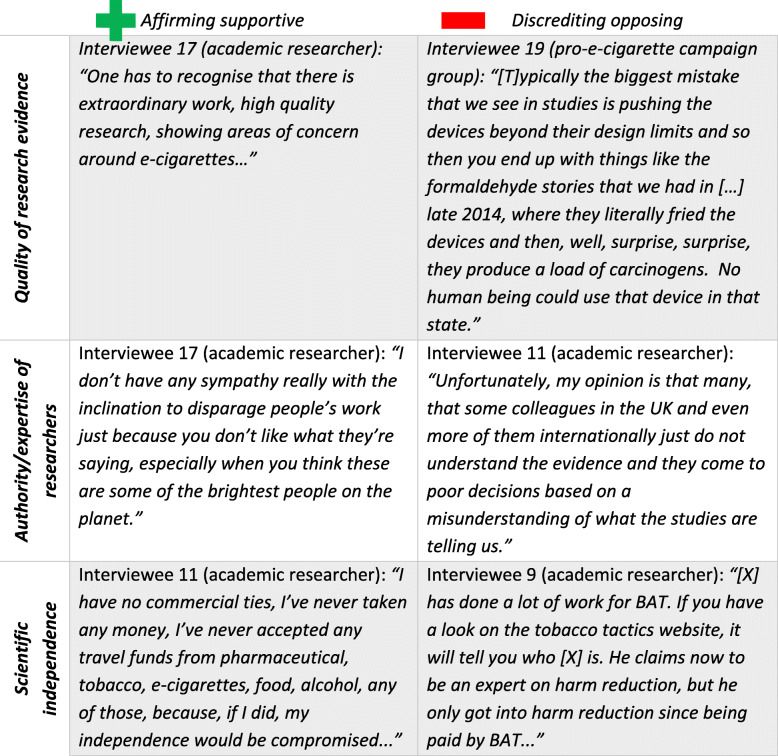
Illustrative examples of ‘boundary work’ in e-cigarette debates.

Such accounts are examples of what Gieryn terms scientific ‘boundary work’, in which participants construct a ‘boundary’ between ‘science’ and ‘non-science’ to reinforce the authority of their preferred perspective [24]. Although Gieryn’s focus was restricted to scientists themselves (and not linked to Hall’s work on ‘policy paradigms’), his account of ‘boundary work’ makes clear why such an approach might be attractive for participants seeking to promote a particular policy paradigm:

‘when the goal is monopolization of professional authority and resources, boundary-work excludes rivals […] by defining them as outsiders with labels such as “pseudo,” “deviant,” or “amateur”’.

In fact, there are so many examples of ‘boundary work’ in action within our data that it is impossible to include them all so, for brevity, Table 2 summarises illustrative data extracts of key types of ‘boundary work’ that we identified.

Overall, while there are occasional references to markers of scientific credibility relating to methods and processes in our data (e.g. one reference to the peer-review process and another highlighting the credibility of systematic reviews compared to single studies), we can also see (in Table 2) that perceptions of scientific authority and credibility are closely intertwined with people’s sense of ‘trust’ which, in turn, appeared to rest heavily on personal-professional relationships and perceptions of independence, expertise and intelligence.

### A temporary lull before post-Brexit battles?

Revisiting our earlier conversations with a sub-set of follow-up interviewees in 2019–2020 suggests that, while e-cigarette products and markets have continued to evolve, the intensity of policy discussions has temporarily abated. The primary explanation for this seemed to be the fact that initial UK regulatory responses have been agreed and enacted. Additionally, interviewees suggested other issues had distracted attention from e-cigarettes, including the emergence of cannabis related products and the need for broader organisational preparations for the UK’s exit from the European Union (and, indeed, some of our potential follow-up interviewees were unable to participate as a result of the need to focus on organisational work relating to the COVID-19 pandemic).

No follow-up interviewee suggested there had been any significant new primary studies, though several noted there had been some major reviews to synthesise available evidence [32]. Interviewees did not suggest any key players within Scotland had made major changes to their policy positions on e-cigarettes but some interviewees suggested that a couple of health actors who had been in the middle of Fig. 2 had shifted somewhat to the left-hand side, as some of their initial concerns had been allayed. In contrast, another noted that a spate of deaths related to vaping in the US [33] may have reignited some concerns about the safety of e-cigarettes. When it comes to the potential for research to address these concerns, several interviewees confirmed their earlier view that only longitudinal research, over a generation, was likely to settle these debates.

Overall, despite some feeling that media outlets continued to present e-cigarettes as divisive, our follow-up interviews suggested research and policy debates were less fraught in 2019–20 than they had been in 2016–17 but that this was due to reduced policy activity, rather than to substantial shifts in the positions of key players or the emergence of any research consensus. For now, e-cigarettes appear to have been accommodated within the UK’s dominant tobacco control policy paradigm, as a means of enabling smokers to reduce or quit smoking. However, it was also clear that post-Brexit regulatory discussions are being positioned as an opportunity for changing the UK’s regulatory approach to e-cigarettes. Indeed, it was notable that interviewees involved in the e-cigarettes industry were already expressing some confidence that post-Brexit changes will enable the further promotion and growth of e-cigarettes in the UK. One interviewee also noted ongoing discussions about the possibility of prescribing e-cigarettes via the National Health Service. All of this suggests that any lull in policy debates about e-cigarettes is likely to be temporary.

## Discussion

In an overview of sociology of science, Pinch and Leuenberger argue that: ‘By studying a scientific controversy one learns something about the underlying dynamics of science and technology and their relations with wider society’, including ‘the normally hidden social dimensions of science’ [34]. In this case, our data suggest that the emergence of e-cigarettes in the UK has contributed to a fracturing of a long-standing public health alliance [18, 19, 27] in ways that imply the actors involved held distinct values and beliefs, which are being illuminated via the new questions, opportunities and challenges that e-cigarettes throw up.

The ensuing debates have clearly been fraught and, like Hawkins and Ettelt [13], we found those who have settled on positions at either end of the regulatory spectrum (particularly those favouring relaxed regulation, many of whom have vested economic interests) tend to employ evidence strategically, in support of their preferred position. For these actors, it seems unlikely that new evidence will inform their preferences regarding the policy response to e-cigarettes. Yet, we also demonstrate that most participants were in an uncertain ‘middle ground’ regarding the regulation of e-cigarettes. For these actors in the middle ground, even though normative outlooks often appeared to be helping them to negotiate the current (limited) evidence-base, most seemed responsive to emerging evidence (indeed, some recounted already having altered their position on e-cigarette regulations in response to new evidence). Our analysis therefore tells a more complex story than that of Hawkins and Ettelt [13]; one that is more optimistic about the role that research evidence is playing in informing people’s positions on regulation. While we agree that potential for evidence to adjudicate areas of clear disagreement or points of principle is limited, our analysis suggests evidence could play an important role in shaping the policy preferences of those whose views about e-cigarettes regulation are less settled.

In theoretical terms, our analysis suggests that Sabatier and Jenkins-Smith’s ACF, which had previously been widely applied to analyses of tobacco policy debates [18, 19, 27], sheds only limited light on this field in the context of e-cigarettes. Although the emphasis that the ACF places on analysing values and interests remains useful, our findings suggest that the sense of antagonism and controversy in Scottish e-cigarettes debates comes not from the existence of two opposing coalitions but from recurrent media simplifications and the work of some actors at either end of the regulatory spectrum to bolster support for their preferred positions. Our analysis suggests that combining Hall’s [23] concept of ‘policy paradigms’ with Gieryn’s [24] notion of ‘boundary work’ is analytically more helpful. We argue the sense of antagonism that marks UK e-cigarette debates, and the behaviours of some actors, arises from the potential for e-cigarettes to usher in a new ‘policy paradigm’ for tobacco. Hence, engaging with e-cigarettes debates means engaging with this broader struggle, making the stakes very high for those who favour one paradigm over the other. Within this, as Hall [23] suggests, we see claims to scientific authority serving as a crucial tool in a battle for authority. Here, we demonstrated the utility of Gieryn’s [24] concept of ‘boundary work’ in understanding how and why actors claim scientific authority for their own position, while trying to undermine the scientific claims of opponents.

Looking to the future, our findings suggest that e-cigarette debates are likely to settle only when a majority of participants in the uncertain ‘middle ground’ agree a more concrete position along the spectrum depicted in Fig. 2. Settling in a more certain ‘middle’ may enable the current paradigm to continue (as appears to be the case for now), while a shift to the left-hand side of Fig. 2 (where most of the actors with an economic interest in e-cigarettes are positioned) seems likely to usher in a harm reduction paradigm, and a shift to the right-hand side (more unlikely, given the small number of actors we identified as openly holding this position), a more neo-prohibitionist paradigm. There is a potential for evidence to play an important role here, given that a large number of actors is occupying the uncertain ‘middle ground’. Viewed optimistically, this suggests research evidence could play a role in overcoming the fragmentation of the UK’s previously strong tobacco control coalition [27]; a fragmentation which is clearly in tobacco industry interests [16].

In the meantime, our follow-up interviews suggest that policy debates are experiencing a temporary lull, during which time there is a reduced spotlight on the ‘scientific showdowns’ we identified. However, these ‘scientific showdowns’ seem likely to re-intensify as new (e.g. post-Brexit) policy debates develop. This means evidence will continue to be constructed and interpreted by actors against a tense backdrop of contrasting claims to scientific authority. The importance attached to scientific authority in public health policy means academics are key actors in these debates and future research should explore not only how actors are deploying evidence in policy debates but also how and why the e-cigarettes evidence-base is being constructed as it is, by whom and to what ends.

## Conclusions

Our data support previous research in identifying policy debates around e-cigarette as highly contentious [13]. Indeed, we show that previously cohesive tobacco control coalitions have unravelled as some actors, with clear preferences regarding policy responses to e-cigarettes, have worked hard to influence those with less fixed views. However, our findings are more optimistic regarding the potential role of evidence in resolving public health fragmentation around e-cigarettes. This is because our results suggest that many participants in Scottish e-cigarette debates have not yet reached a settled position on regulating e-cigarettes and instead occupy an uncertain ‘middle ground’ which, crucially, appears to be responsive to emerging evidence.

## Supplementary Information

**Additional file 1.** Generic version of the main interview schedule, which lists the questions we put to interviewees (NB as is usual with semi-structured, qualitative interviews, the ordering and specific wording of the questions was sometimes tweaked to allow the interviewer to maintain the flow of the conversation and ensure questioning was responsive to information the interviewee provided).

**Additional file 2.** Generic version of the follow-up interview schedule, used in the follow-up interviews conducted August 2019–March 2020.

## Data Availability

The policy consultations analysed in this paper are publicly available documents which can be accessed here: https://www.gov.scot/publications/consultation-electronic-cigarettes-strengthening-tobacco-control-responses/ and here: http://www.parliament.scot/parliamentarybusiness/CurrentCommittees/91459.aspx. The participants who took part as interviewees consented to participate on the basis that: (i) they would remain anonymous; and (ii) the full data would only be available to the research team and only anonymised extracts would be published (since publishing the full transcripts would be likely to reveal interviewees’ identities, at least to others working in this field). All research team members have seen the full interview transcripts.

## Abbreviations

ACF: Advocacy Coalition Framework
NHS: National Health Service (UK)
