# Supplementary material for: Evidence use in E-cigarettes debates: scientific showdowns in a ‘wild west’ of research
Source: BMC Public Health. 2021 Feb 16;21:362. doi: 10.1186/s12889-021-10396-6 (PMC7884966; doi:10.1186/s12889-021-10396-6)
Supplement: Supplementary file 1 — Additional file 1. Generic version of the main interview schedule, which lists the questions we put to interviewees (NB as is usual with semi-structured, qualitative interviews, the ordering and specific wording of the questions was sometimes tweaked to allow the interviewer to maintain the flow of the conversation and ensure questioning was responsive to information the interviewee provided). [file 12889_2021_10396_MOESM1_ESM.docx]

**Interview topic guide**

(This topic guide will be tailored slightly in order to fit with the respective interviewee)

| Interviewer: | Date of Interview: |
| --- | --- |
| Interview No: | Organisation/Role: |
| Interview ID: | Descriptor used: |
|  |  |
| Introduction  This project investigates stakeholder engagement in emerging policy debates on e-cigarette regulation. We are interested in the views of a variety of stakeholders who are interested in, or involved in the development of UK e-cigarette regulation.  Views of the e-cigarette regulation and the policy process (Narrative part)  Could you tell me about your involvement in/views of the e-cigarette policy process?  What were the issues that were most contentious?  Who were the most vocal stakeholders in the e-cigarette debate? How did stakeholders influence the policy process?  What is your understanding of current e-cigarette regulation? What do you perceive to be the biggest issues with regard to e-cigarette regulation at the moment?  What do you think about existing e-cigarette regulation? How would you regulate e-cigarettes if you were in charge? (Or don’t support regulation) Why would you take this approach?  There has been some debate around vaping in public places. What are your thoughts on this?  (Also ask about views on advertising and protecting minors, prompt on content regulated through TPD, retail register, if interviewee has not mentioned).  (Using further prompts if necessary: Scottish Consultation on e-cigarette regulation, introduction of the European Tobacco Products Directive in May 2016, recent regulation to protect minors, etc.) | |
| Reasons for political engagement (Semi-structured part)  How much of a priority is e-cigarette regulation for you/your organisation?  What are the reasons you are/the organisation you are representing is interested in e-cigarette policy/regulation?  What is your/your organisation’s position on e-cigarette regulation?  Which aspect of e-cigarette policy/regulation are you most interested/involved in? Why?  To what extent is/has your/your organisation’s view being/been taken into account?  In terms of influencing e-cigarette policy/regulation, how do you/did you make sure your position is/was taken into account? | |
|  | |
| Beliefs, knowledge and evidence use  What do you think are potential benefits and harms of e-cigarettes?  Where do you get information about e-cigarettes from?  What kind of information do you think is missing?  Who do you talk to if you need information? Which information sources do you access? | |
| Collaboration in e-cigarette regulation  Are you aware of any stakeholders, including your own organisation and others, working together to influence e-cigarette policy/regulation?  Why, do you think, did/do these organisations co-operate?  Who do you collaborate with? Who would you NOT collaborate with? What are the reasons why/why not?  Perception of commercial sector engagement in e-cigarette regulation  What do you think are the advantages and disadvantages of involving commercial actors in the development of e-cigarette policy?  Do you see problematic areas/conflicts of interest between commercial actors and their involvement in e-cigarette policy debates? Why? Which ones? Can you elaborate?  How do you think policy makers should handle commercial actor engagement in e-cigarette regulation? | |
| Close of interview  Would you like to be informed about the results of my study?  Do you have any thoughts on **who else I should interview** regarding this topic?  Is there anything that we have missed?  Do you have any questions? | |
