# Supplementary material for: Evidence use in E-cigarettes debates: scientific showdowns in a ‘wild west’ of research
Source: BMC Public Health. 2021 Feb 16;21:362. doi: 10.1186/s12889-021-10396-6 (PMC7884966; doi:10.1186/s12889-021-10396-6)
Supplement: Supplementary file 2 — Additional file 2. Generic version of the follow-up interview schedule, used in the follow-up interviews conducted August 2019–March 2020. [file 12889_2021_10396_MOESM2_ESM.docx]

**Interview topic guide for follow-up interviews**

(This topic guide will be tailored slightly in order to fit with the respective interviewee)

| Interviewer: | Date of Interview: |
| --- | --- |
| Interview No/ID: | Organisation/Role: |
| **Introduction**  This project investigates stakeholders’ changing perceptions and views on policy debates on e-cigarette regulation. We are interested in following up with a variety of stakeholders who are interested in UK e-cigarette regulation, to explore how their views have changed. This interview will use some of the findings from earlier interviews and will investigate changes in the debate on e-cigarette regulation. | |
| **Changing views of e-cigarette regulation**  When you were interviewed about this topic a couple of years ago (ADD DATE PERSONALISED TO INTERVIEWEE), we had some discussion about e-cigarettes, the related evidence-base and about policy responses). I’m keen to know how you feel debates have moved on since then.  What do you feel have been the **biggest changes in e-cigarette debates** since we last spoke?  Many of the interviewees we spoke to suggested that debates about e-cigarettes were extremely fraught in Scotland and the wider UK. How would you say has the nature of debates about e-cigarettes evolved since we last spoke? Have your own positions within (or views about) these debates changed since then? Why has it changed?  What do you feel have been the biggest changes to e-cigarettes in terms of the **technology** and the **market** since we last spoke? Have these changes influenced how you feel about e-cigarettes?  Would you say there have been any important policy developments/changes relating to e-cigarettes? If so, what do you think about these developments?  What do you feel are the key debates / questions relating to e-cigarettes that currently exist?  How much of a priority do you feel e-cigarettes are for your organisation/group/team at the moment? (in comparison to last time we spoke/changes) Why do you think this is? | |
| **Changes in engagement in e-cigarette regulation**  Based on your experiences of engaging in e-cigarette policy discussions in Scotland/the UK, who would you say has been particularly influential in e-cigarette debates over the last three years? Why these ones? Has it changed in the last two- three years? | |
| **Changes in commercial sector engagement in e-cigarette regulation**  Do you think there have been any important changes to the commercial sector for e-cigarettes over the past 2-3 years?  Would you say there have been any noticeable changes with how the commercial sector has been engaging in research and policy debates about e-cigarettes over the last couple of years? If so, how has this changed and why do you think it has changed? | |
| **Changes in beliefs, knowledge and evidence use**  How would you say the **research evidence** relating to e-cigarettes has progressed over the last 2-3 years? Are you aware of any particularly important developments in the evidence-base relating to e-cigarettes (or their regulation)?  How do you tend to find out about new research evidence relating to e-cigarettes?  Several of the people we interviewed last time said they felt that the quality of the available evidence relating to e-cigarettes was not very good (at that time). What do you feel the quality of the available evidence is now, a few years later?  When you come across evidence relating to e-cigarettes, how do you attempt to assess its **credibility/trustworthiness**?  Are you hopeful that research will help resolve key debates / questions relating to e-cigarettes? If so, how and in what kind of timescale? If not, why not?  Several people we spoke to last time suggested they felt it was important to consider who is **funding** research on e-cigarettes. Do you have a sense of who is funding research in this area and whether this is likely to impact on the findings/conclusions? Who do you think should be funding research on this topic?  What role do you feel the **media** plays in research and policy debates about e-cigarettes? | |
| **Close of interview**  To wrap up, thinking about the future, what is the **key unanswered question** about e-cigarettes and their regulation that you think researchers should be trying to address?  Is there anything that we have missed or anything you’d like to add?  Do you have any questions? | |
